# *Junonia coenia* Densovirus (JcDNV) Genome Structure

**DOI:** 10.1128/genomeA.00591-13

**Published:** 2013-08-08

**Authors:** Hanh T. Pham, Oanh T. H. Huynh, Françoise-Xavière Jousset, Max Bergoin, Peter Tijssen

**Affiliations:** INRS-Institut Armand-Frappier, Laval, QC, Canadaa; Laboratoire de Pathologie Comparée, Université Montpellier II, Montpellier, Franceb

## Abstract

The sequence of *Junonia coenia* densovirus was the first densovirus genome sequence published, but the first published sequence contained incomplete inverted terminal repeats and ambiguous nucleotides or indels leading to an incorrect map of the open reading frames. Our sequencing of clones of the complete genome demonstrated that this virus is closely related to other viruses in the *Densovirus* genus.

## GENOME ANNOUNCEMENT

The common buckeye (*Junonia coenia* [Hübn.]) is a butterfly in the *Nymphalidae* family. A nonoccluded, small virus was isolated from caterpillar cadavers (1) and subsequently characterized as a densovirus (2). This *Junonia coenia* densovirus (JcDNV) was kindly provided to us by T. W. Tinsley (NERC Institute of Virology, Oxford). Its restriction map showed a close relatedness to the GmDNV densovirus from *Galleria mellonella* (3). An infectious JcDNV genome was cloned (pBRJ) and sequenced (4, 5).

Related densoviruses have been isolated, cloned, and sequenced, e.g., from *Galleria mellonella* [GmDNV (6)], *Mythimna loreyi* [MlDNV (7)], *Helicoverpa armigera* [HaDNV (8)], and *Pseudoplusia includens* [PiDNV (9)]. Compared to the published genome sequences of this group, the published JcDNV sequence had a different genome orientation, incomplete inverted terminal repeats (ITRs), and a different map of open reading frames (ORFs). In addition, there are several ambiguous sequences in the reported genome sequence of JcDNV (GenBank accession number NC_004284).

Here, the entire JcDNV genome was extracted from the same virus stock as that used to produce pBRJ, recloned, and sequenced and compared to the genome sequence of pBRJ. The separately encapsidated, complementary DNA strands reannealed upon extraction. The central 5.45-kb part of the genome sequence, after BamHI digestion, was cloned into pBluescript KS. The rest of the inverted terminal repeats (ITRs) were obtained by PCR from gel-purified 2.4- and 3.6-kb dsDNA fragments after digestion with NdeI. DNA was heated up to 95°C for 5 min, and 10 ng of each fragment was used for ligation with a 5P-ACGCAAGTACCGTGGTACCATGGATCCGG-3C3 adapter, including 1% DMSO, 1 mM hexamine cobalt chloride, and 10% polyethylene glycol (PEG) (final concentrations) and T4 RNA ligase (NEB), and then incubated overnight at room temperature. The DNA was then precipitated and eluted in 70 µL of sterile water. Five microliters was used for 25 µL of PCR, including 6% DMSO, 1.3 M betaine, 50 µM 7-deaza-dGTP, CTTCGGATCCTCTCCATCATC, and CCGGATCCATGGTACCACGGTACTTGCGT as specific and adapter primers, respectively, and Phusion High-Fidelity DNA polymerase (98°C, 3 min; 25 cycles of 98°C 10 s, 65°C 20 s, and 72°C 20 s; elongation, 72°C, 5 min). Amplicons were cloned into a pGEMT-easy TA vector (Promega) and transformed into Sure cells. Two complete clones were sequenced in both directions and pBRJ was sequenced at locations of discrepancies, using Sanger’s method and the primer-walking method as described before (6). The contigs were assembled by using the CAP3 program (http://pbil.univ-lyon1.fr/cap3.php/) (10).

The 6,032-nucleotide (nt) JcDNV genome contained 547-nt-long, nearly identical ITRs, the only differences being 396G, 537G, and the two TATA boxes (539-TATAAAT for the NS-gene cassette and 5488-TATATAA for the VP-gene cassette). The typical terminal Y-shaped hairpins of 130 nt contained two orientations at sequences 51 to 80 and 5953 to 5982, “flip” and its reverse complement orientation “flop.” The ambisense JcDNV genome sequence was 83 to 87% identical to those of other viruses in the *Densovirus* genus. The ORFs, in contrast to the previous entry in GenBank, were conserved with members of the *Densovirus* genus, as were the splicing sites (11) with those identified for GmDNV (6) and MlDNV (7). Also, sequencing errors, gaps, and ambiguous nts were corrected and the ITRs completed. JcDNV contained the typical NS-1 helicase superfamily III and VP phospholipase A2 (12) motifs observed in other parvoviruses.

### Nucleotide sequence accession number.

The GenBank accession number of JcDNV is KC883978.
